# Family history of substance use disorders: Significance for mental health in young adults who gamble

**DOI:** 10.1556/2006.2020.00017

**Published:** 2020-06-05

**Authors:** Jon E. Grant, Samuel R. Chamberlain

**Affiliations:** 1 Department of Psychiatry & Behavioral Neuroscience, University of Chicago, Chicago, IL, USA; 2 Department of Psychiatry, University of Cambridge, Cambridge, UK; 3 Cambridge and Peterborough NHS Foundation Trust (CPFT), Cambridge, UK

**Keywords:** family history, substance use, addiction, cognition

## Abstract

**Background:**

Although family history of psychiatric disorders has often been considered potentially useful in understanding clinical presentations in patients, it is less clear what a positive family history means for people who gamble in the general community. We sought to understand the clinical and cognitive impact of having a first-degree relative with a substance use disorder (SUD) in a sample of non-treatment seeking young adults.

**Methods:**

576 participants (aged 18–29 years) who gambled at least five times in the preceding year undertook clinical and neurocognitive evaluations. Those with a first-degree relative with a SUD were compared to those without on a number of demographic, clinical and cognitive measures. We used Partial Least Squares (PLS) regression to identify which variables (if any) were significantly associated with family history of SUDs, controlling for the influence of other variables on each other.

**Results:**

180 (31.3%) participants had a first-degree family member with a SUD. In terms of clinical variables, family history of SUD was significantly associated with higher rates of substance use (alcohol, nicotine), higher rates of problem gambling, and higher occurrence of mental health disorders. Family history of SUD was also associated with more set-shifting problems (plus higher rates of obsessive-compulsive tendencies), lower quality of decision-making, and more spatial working memory errors.

**Conclusions:**

These results indicate that gamblers with a first-degree family member with a SUD may have a unique clinical and cognition presentation. Understanding these differences may be relevant to developing more individualized treatment approaches for disordered gambling. Compulsivity may be important as a proxy of vulnerability towards addiction.

## Introduction

Family history of substance use disorders (SUDs) has been examined for the past forty years, most often in the context of understanding phenomenological differences in those adults with alcoholism who have or do not have a family history of addictions (Latcham, 1985; Penick, Nickel, Powell, Bingham, & Liskow, 1990), identifying predictive factors that may or may not result in treatment differences in those with alcoholism (Drake et al., 1995), understanding cognitive and biological differences seen in neuroimaging of alcoholics (de Wit & McCracken, 1990; Krystal et al., 2003; Muller et al., 2015; Schaeffer, Parsons, & Yohman, 1984), and identifying those at risk for developing SUDs (Barnow, Schuckit, Lucht, John, & Freyberger, 2002; Beseler, Aharonovich, Keyes, & Hasin, 2008; Cloninger, Bohman & Sigvardsson, 1981; Dawson, Harford, & Grant, 1992; Goodwin, 1983; Harrington, Robinson, Bolton, Sareen, & Bolton, 2011; Schuckit and Duby, 1982).

Other, albeit limited, research has suggested that a family history of SUDs may be an important information in terms of how it impacts other psychiatric symptoms. For example, an early study of patients with bulimia nervosa found that those with a family history of drug abuse were more likely to have experienced drug abuse problems themselves, to have a history of having been overweight, and report more family disruption (Mitchell, Hatsukami, Pyle, & Eckert, 1988). Another study found that people with trichotillomania or skin picking disorder, who also had a family history of SUDs, exhibited more severe forms of illness, more depressive symptoms and higher rates of co-occurring ADHD, than those with the identical disorders but no family history of addictions (Redden, Leppink, & Grant, 2016). These studies suggest that family history of SUDs may have important clinical associations beyond their predictive effects of a future SUD.

Gambling is a commonplace activity across much of the world, and the majority of individuals who gambling do so recreationally, without developing disordered gambling. Disordered gambling encompasses a number of features, conceptually derived largely from prior work in substance use disorders, including escalation over time, difficulty cutting back, neglecting other areas of live, and functional impairment (Hodgins, Stea, & Grant, 2011). Gambling Disorder is the only behavioral addiction currently listed in the Diagnostic and Statistical Manual Version 5 (DSM-5) category of Substance-Related and Addictive Disorders (American Psychiatric Association, 2013). Gambling exists along a continuum from endorsement of none to endorsement of all Gambling Disorder diagnostic criteria. Research indicates that endorsement even of a couple of diagnostic criteria can be sufficient to be associated with marked functional impairment (Chamberlain, Stochl, Redden, Odlaug, & Grant, 2017). Despite gambling symptoms being nosologically related to substance addictions in the DSM-5, surprisingly little research has examined the impact of family history of SUDs on the clinical and cognitive presentation of people who gamble. Such examination could have implications for tailoring treatments for disordered gambling.

One way to understand how a family history of SUDs might impact gambling is to see it as a potential clinical marker for underlying relational/cognitive/genetic/neurobiological issues that are not beholden to diagnostic boundaries. Insights can be gleaned from findings other than gambling. For example, one recent study performed a longitudinal functional magnetic resonance imaging in 43 children of alcoholic families (family history positive) and 30 children of controls (family history negative) using a go/no-go task. The study found that differences in response inhibition circuitry were visible in family history positive individuals during childhood and into adolescence (Hardee, Weiland, & Nichols, 2014). A recent review examined the neurobiological phenotypes present in youth and adults with positive family histories for alcoholism by describing findings across neurophysiological and neuroimaging studies. The review found that individuals with positive family histories differed from their peers in amygdalar, hippocampal, basal ganglia, and cerebellar volumes, with mixed directions of effect (Cservenka, 2016). In addition, functional magnetic resonance imaging studies have found altered inhibitory control and working memory-related brain response in youth and adults with positive family histories, suggesting neural markers of executive functioning may be related to increased vulnerability for developing alcohol use disorders in this population (Cservenka, 2016). One large Internet-based assessment of mental health in college students (*n* = 6,032–7,169) found that a family history of alcohol and drug problems, in addition to being associated with increased alcohol consumption, were weakly and positively associated with openness, extraversion and neuroticism, and modestly associated with impulsivity (Kendler et al., 2015). Although such studies suggest that these findings from neuroimaging and cognitive profiles may be contributing factors for subsequent substance use problems, one could also imagine that these cognitive/imaging findings might predispose to disordered gambling, given the neurobiological overlap with SUDs (Grant & Chamberlain, 2019). In a recent consensus statement, it was reasoned that different mechanisms may be involved in disposition towards addictions, whereas other variables may be more important in terms of chronicity (Yucel et al., 2019). Overall, there was consensus that cognitive abnormalities reflecting reward dysfunction, disinhibition, and action selection were likely to be important in terms of addiction vulnerability and chronicity; whereas other aspects (such as habit and compulsivity) were hypothesized to be relatively unimportant for vulnerability (Yucel et al., 2019).

Understanding of gambling “subtypes” and how to classify people with gambling problems is highly relevant from a clinical perspective, and family history may be one way to more effectively categorize people. Because research suggests that young adulthood may represent a vulnerable time to develop gambling problems (Welte, Barnes, Tidwell, & Hoffman, 2008), and that different developmental, psychosocial, and cognitive pathways may underlie the development of problem gambling in young adults (Dowd, Keough, Jakobson, Bolton, & Edgerton, 2019; Grant, Chamberlain, Schreiber, Odlaug, & Kim, 2011), young adults who gamble may be ideally situated for study before other confounding variables (i.e. greater brain dysfunction and comorbidities) take effect.

Although possibly predictive of future addictive behavior, existing data on family history provide only limited information as to what, if anything, these types of familial associations may mean for gamblers in the general population, as opposed to specific groups of patients recruited from clinical settings. Therefore, understanding differences between the gambling individuals with positive and negative family histories of SUDs may be important in order to identify potentially clinical and cognitive subtypes and improve neurobiological models and treatment. The purpose of this study was to investigate whether young adults who gamble with a first-degree relative with a SUD had a different clinical and cognitive presentation than those without, and whether analysis of different families' histories had any clinical relevance.

## Methods

### Subjects

576 young adult participants (aged 18–29 years) were enrolled from a study of impulsivity in young adults. Study inclusion criterion was participants had gambled at least five times in the past year and that they were able to be interviewed in person. The only exclusion criterion was the inability to understand and consent to the study. Participants were recruited in the Minneapolis and Chicago metropolitan areas using media advertisements. Participants were informed that there was no treatment component to this study. Each participant received a $50 gift card to an online store as compensation. The assessment took approximately 4 h in total, with cognitive testing being approximately 45 min long. Participants were free to take breaks as needed during the assessment visits.

### Assessments

Demographic variables, including age, gender, and highest level of education completed, were recorded for all participants. Subjects received a psychiatric evaluation, which included the Mini International Neuropsychiatric Inventory (MINI) (Sheehan et al., 1988) (a clinician-administered psychiatric interview that evaluates for major depressive disorder, panic disorder, generalized anxiety disorder, eating disorders, and others); the Minnesota Impulsive Disorders Interview (MIDI) (which screens for impulse control disorders, including compulsive buying, kleptomania, trichotillomania, skin picking disorder, pyromania, intermittent explosive disorder, compulsive sexual behavior, and binge eating disorder) (Chamberlain and Grant, 2018; Grant, 2008); Structured Clinical Interview for Pathological Gambling (SCI-PG) (Grant, Steinberg, Kim, Rounsaville, & Potenza, 2004) adapted for DSM-5; the Barratt Impulsiveness Scale (BIS-11) (a self-report questionnaire, was employed to quantify impulsive personality; Patton, Stanford, & Barratt, 1995; Stanford et al., 2016); the Padua Inventory (PADUA) (questionnaire consisting of 39 items, assessed common obsessive and compulsive phenomena; Sanavio, 1988); Quality of life was measured using the Quality of Life Inventory (QOLI) (Frisch, Cornell, & Villanueva, 1992).

In addition to paper-pencil measures, participants underwent selected cognitive tests from the Cambridge Neuropsychological Test Automated Battery after the clinical interview. Tasks were administered in a fixed order. Study subjects completed the following cognitive tasks in a quiet room using a touch screen computer under the guidance of a trained assessor:

#### Intra-Extra Dimensional Set Shift Task (IED)

This task examines cognitive flexibility.

Subjects are presented with four boxes: two contain pink shapes and two are blank. Using a rule set by the computer, subjects are notified that one of the displayed shapes is correct and the other is incorrect. Individuals must learn this rule and then select the correct shape in as many trials as possible. Once the subject chooses a number of correct shapes the computer switches the rule to introduce a new “correct” shape. The subject must adapt; this is the intra-dimensional set shift. Following this portion of the task, the computer introduces a set of white shapes overlaying the pink shapes. The new correct shape is one of the white shapes. Again, the subject must identify the correct shape as chosen by the computer. This addition of stimuli is the extra-dimensional set shift (ED). The number of total errors throughout the task was the outcome measure of interest (Owen, Roberts, Polkey, Sahakian, & Robbins, 1991).

#### Stop Signal Response Task (SSRT)

This task measures response inhibition. Subjects are presented with a series of directional arrows that appear one at a time on the screen. The subject must immediately press the corresponding arrow computer key matching the direction of the arrow as fast as they are able. When a buzzer sounds after the directional arrow is displayed the subject must resist pressing the computer key. The estimated time it takes for the subject to suppress the already triggered response when the buzzer sounds is calculated as the “stop signal reaction time” (Aron et al., 2007).

#### Cambridge Gambling Task (CGT)

The CGT examines decision-making. During each trial subjects are presented with ten blocks, a portion of which are red and a portion of which are blue. A token randomly resides under one of these ten boxes. Subjects must decide if they think the token resides under a red or blue box. After a decision is made they are given an opportunity to bet a certain amount by pressing a box showing decreasing values on the screen. After a time, the box shows incrementing or decrementing values and the subject must again decide how much they want to bet. The outcome measures of interest were the total proportion of points gambled, the quality of decision-making, and risk adjustment (Rogers et al., 1999).

#### Spatial Working Memory Task (SWM)

The SWM tests the ability to remember spatial information and to use working memory in that process. Similarly to the OTS, PIU may be characterized by impaired working memory performance. Colored boxes are presented on a screen, one containing a blue box in it. Subjects click the boxes in order to find the blue box. Once they find it, they can use process of elimination to find all of the other blue boxes until a column on the right of the screen is filled. The number of boxes in each trial increases over time. SWM total errors is the number of times the subject clicks a box that is known not to contain a blue box (Owen, Downes, Sahakian, Polkey, & Robbins, 1990).

### Family history assessment

We undertook the family history method where the proband is asked about psychiatric and substance use problems in their relatives, despite its methodological limitations (Andreasen, Endicott, Spitzer, & Winokur, 1977; Kendler et al., 1991). Participants were asked about the presence of SUDs (which included alcohol and drug use disorders, but not nicotine) in all first-degree relatives. Substance use disorders were defined as the chronic use of drugs or alcohol resulting in either noticeable social and occupational dysfunction or the need for a twelve-step program or formal treatment. All information about relatives came from the proband. No direct evaluations of the first-degree relatives were performed.

### Data analysis

Differences in demographic, clinical, and cognitive variables between the groups were identified using analysis of variance (ANOVA). Results were cross-checked using non-parametric tests where normality assumptions were violated. Statistical significance was defined as *P* < 0.05, Bonferroni corrected for the number of multiple comparisons.

In order to identify variables associated with family history of SUDs, whilst controlling for inter-relationships between such variables, we used the powerful statistical method of Partial Least Squares regression (hereafter referred to as “PLS”). PLS is a versatile multivariate approach to data modeling that analyses relationships between one set of variables (*X*) and another set of variables (*Y*) by means of fitting one or more latent components (Wold, Sjostrom, & Eriksson, 2001). Unlike standard regression, PLS is robust to violations of normality assumptions and to item cross-correlations. Hence PLS is ideally suited to the current dataset. Candidate *X* variables in the PLS model were the demographic/clinical/cognitive measures, and the *Y* variable of interest was family history of SUDs. By convention, and for computational reasons, the *X* matrix is usually the larger set of variables; we do not mean to suggest by this that *X* causes *Y*. Rather, we used PLS as a valuable tool to understand the relationships between these two sets of variables.

PLS modeling was conducted using JMP Pro software. The PLS model was fitted using leave-one-out cross-validation (non-linear iterative partial least squares, NIPALS, algorithm), and the optimal model was identified based on minimizing predictive residual sum of the squares (PRESS) per convention. Only *X* variables with a Variable Importance Parameter (VIP) >0.8 were retained in the model, in line with recommendations for PLS modeling. *X* variables significantly contributing to the model (i.e., explaining significant variance in current compulsive and impulsive problem behaviors) were identified on the basis of 95% confidence intervals for bootstrap distribution of the standardised model coefficients not crossing zero (*N* = 1,500 bootstraps; *P* < 0.05).

### Ethics

The Institutional Review Board of the University of Chicago approved the study and the consent statement. The authors assert that all procedures contributing to this work comply with the ethical standards of the relevant national and institutional committees on human experimentation and with the Helsinki Declaration of 1975, as revised in 2008.

## Results

Of the 576 young adult gamblers, 180 (31.3%) reported a first-degree family member with a SUD. Comparisons between the two groups on the variables of interest are summarized in Table 1. For demographic variables, family history of SUDs was significantly associated with older age, female gender, non-Caucasian racial-ethnic group, and lower quality of life.

**Table 1.** Demographic, clinical, and cognitive differences between young adult gamblers with and without a family history of substance use disorders

Measure	Total sample Family history SUDs		*F*	*P* (uncorr)
	No	Yes		
	*N* = 396	*N* = 180		
Age, years	21.8 (3.5)	23.4 (3.5)	27.23	<0.0001∗
Gender, male *N* [%]	277 [70.0%]	100 [55.5%]	11.1391	0.0008∗
Education level	3.2 (0.8)	3.1 (0.9)	1.6165	0.2041
Race, Caucasian, *N* [%] #	304 [76.8%]	110 [61.8%]	35.544	0.0001∗
Quality of life *t*-score	47.1 (11.4)	43.3 (12.6)	12.7391	0.0004∗
Amount lost to gambling, past year, United States dollars	960 (3,320)	2,171 (4,972)	11.8388	0.0006∗
Alcohol consumption, times/week	1.3 (1.4)	1.7 (1.5)	8.6909	0.0033
Nicotine consumption, packs per day	0.08 (0.23)	0.23 (0.36)	33.8947	<0.0001∗
SCI-PG score	0.8 (1.6)	2.2 (2.5)	44.7673	<0.0001∗
Any MINI mental disorder, *N* [%]	124 [31.3%]	86 [48.0%]	14.649	0.0001∗
Any MIDI disorder, *N* [%]	35 [8.8%]	26 [14.4%]	3.92	0.0477
Padua obsessive-compulsive inventory total score	16.4 (15.3)	22.2 (21.8)	13.0813	0.0003∗
BIS total score	64.1 (11.5)	66.6 (11.5)	5.7978	0.01464
IED total errors	22.3 (21.7)	31.1 (26.4)	17.6	<0.0001∗
SST SSRT, ms	179.2 (60.7)	188.3 (71.3)	2.4959	0.1147
CGT proportion bet	0.54 (0.14)	0.54 (0.14)	0.0148	0.9033
CGT rational decision-making	0.95 (0.08)	0.93 (0.10)	8.4042	0.0039
CGT risk-adjustment	1.66 (1.21)	1.30 (1.18)	11.0984	0.0009∗
SWM total errors	16.8 (17.8)	22.5 (18.7)	12.5056	0.0004∗

∗ *P* < 0.05 significant group difference with Bonferroni correction (threshold 0.05/21 = 0.0024). Statistical tests are ANOVA for continuous variables, and Likelihood Ratio tests for categorical measures. # presented in binary form for simplicity but all levels analyzed.

Abbreviations: SCI-PG = total symptoms endorsed from Structured Clinical Interview for Gambling Disorder; MINI = Mini-International Neuropsychiatric Inventory; MIDI = Minnesota Impulse Disorders Interview; BIS = Barratt Impulsivity Scale; IED = Intra-Dimensional/Extra-Dimensional Set-Shift Task; SST = Stop-Signal Task; SSRT = Stop-Signal Reaction Time; CGT = Cambridge Gamble Task; SWM = Spatial Working Memory Task.

For clinical variables, family history of SUDs was significantly associated with more money lost to gambling in the past year, higher SCI-PG scores, greater nicotine consumption, higher occurrence of one or more MINI mental disorders, and higher Padua obsessive-compulsive scores.

For cognitive variables, family history of SUDs was significantly associated with more IED total errors (adjusted), less risk adjustment on the CGT, and more SWM errors.

**Figure 1.**
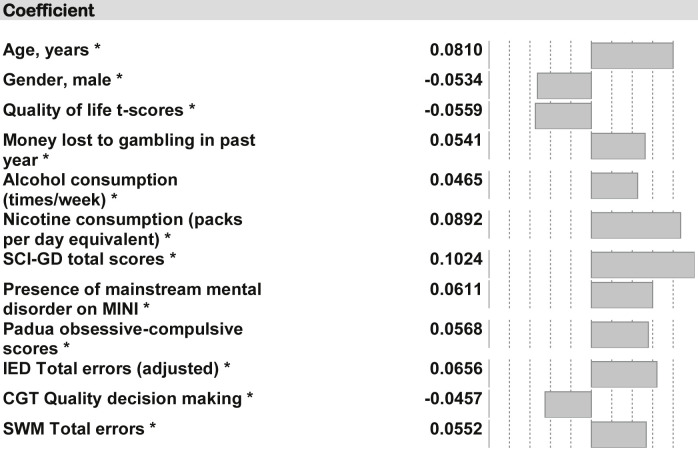
Model coefficients for centered and scaled data, in the PLS model relating the variables below (*X*) to family history of substance use disorders (*Y*). * *P* <0.05 statistically significant by bootstrap. Abbreviations: SCIPG = total symptoms endorsed from Structured Clinical Interview for Gambling Disorder; MINI = Mini-International Neuropsychiatric Inventory; IED = Intra-Dimensional/Extra-Dimensional Set-Shift Task; CGT = Cambridge Gamble Task; SWM = Spatial Working Memory Task

In Partial Least Squares (PLS) analysis, an optimal one-factor model was identified that accounted for 22.7% of variance in the demographic/clinical/cognitive variables, and 13.8% of variation in family history of addiction status. Demographic, clinical, and cognitive variables significant in the model (*P* < 0.05, bootstrap) are shown in Fig. 1. Family history of SUDs was significantly related, in the PLS model, to older age, female gender, lower quality of life, more money lost to gambling in the past year, more gambling symptoms, greater alcohol use, higher cigarette use, and higher presence of mainstream mental disorders on the MINI. Family history of SUDs was also significantly associated with more IED errors, worse quality of decision-making on the CGT, and more working memory errors on the SWM.

## Discussion

This study examined demographic, clinical, and cognitive associations with family history of substance use disorders (SUDs), in a large sample of non-treatment seeking, community-dwelling gamblers. The study found that family history of SUDs had a number of important associations in gamblers. Here, we focus on those that were significant in Partial Least Squares regression modeling, because this statistical approach effectively controls for the potentially confounding influence of variables on each other, indicating that the associations were robust even accounting for the influence of other variables.

For the demographic measures, family history of SUDs was associated with older age and higher likelihood of female gender. The finding of gender differences is provocative but not new. In fact, an early study by Petry and colleagues found that women with a positive family history of alcoholism had higher discount rates than women with a negative family history and could be suggestive of different mechanisms by which risk is transmitted between genders (Petry, Kirby, & Kranzler, 2002).

For the clinical measures, family history of SUDs was associated with greater substance use (alcohol, nicotine/smoking), and higher rates of disordered gambling problems (both in terms of the number of gambling disorder symptoms endorsed, and the amount of money lost to gambling in the past year). The likely explanation for this is that addiction tends to run in families, likely due to the influence of genetic, cognitive, and environmental factors. Prior research in twins indicates that substance use and gambling disorder have shared genetic contributions (Huggett, Winiger, Corley, Hewitt, & Stallings, 2019; Lobo & Kennedy, 2009; Slutske, Ellingson, Richmond-Rakerd, Zhu, & Martin, 2013). In terms of common environmental mediators that may contribute, observing one's parents having an addiction could lead to “modelling” whereby offspring are more likely to also develop similar problems over the course of time. Family history of SUDs was also associated with lower quality of life. In terms of mental disorders, in general the likelihood of any mental disorder (including depression, anxiety, and substance use related) was higher in those with a family history of SUDs. Presence of impulse control disorders on the MIDI was not significantly related to family history of SUDs, but these conditions were relatively uncommon, which would have limited the ability to detect such associations.

Turning to the cognitive variables, family history of SUDs was associated with worse set-shifting (IED task), lower quality of decision-making (CGT task), and more working memory errors (SWM task). Worse set-shifting is in keeping with also finding that family history of SUDs was linked with higher compulsive tendencies on the Padua inventory, indicating that a predilection towards a more rigid cognitive style may tend to run in families and predispose towards developing compulsive clinical problems. Deficits on the IED task are a common finding in compulsive disorders such as OCD, and gambling disorder (Leppink, Redden, Chamberlain, & Grant, 2016), and also are found in clinically asymptomatic first-degree family members of OCD patients (Chamberlain, Blackwell, Fineberg, Robbins, & Sahakian, 2005; Chamberlain et al., 2007a; van Timmeren, Daams, van Holst, & Goudriaan, 2018). In terms of quality of decision-making, this measure has previously been found to be impaired even in the relatively early stages of disordered gambling, when subjects endorse some diagnostic criteria but insufficient for a full diagnosis (Grant et al., 2011). Working memory constitutes another distinct aspect of executive functioning, and has previously been found to be impaired in some OCD studies, especially when using relatively demanding tasks (Harkin & Kessler, 2011) such as the SWM (Chamberlain et al., 2007b). Findings using SWM in gambling disorder are mixed (Clark, 2014).

In terms of cognitive processes that may be involved in different stages of addiction (Yucel et al., 2019), if one views family history of addiction as a proxy for vulnerability, our data indicate that compulsivity (as indexed by the Padua inventory and set-shift task) may be very important even in the earlier stages of addiction, i.e. in terms of rendering one vulnerable to developing an addiction. This is potentially important because it runs counter to the prevailing current view of experts, who felt compulsivity would only be important in terms of chronicity but not vulnerability (Yucel et al., 2019). Thus, future work should evaluate whether trans-diagnostic markers of compulsivity in fact are important in addiction vulnerability in general, ideally using longitudinal studies. By tradition, the main focus has been on impulsivity rather than compulsivity.

If family history of SUDs is a useful clinical subtype of young adult gamblers, can we improve treatments using this subtype? One approach would be to use this information to identify vulnerable young adults and then test early interventions. For example, if a family history of SUDs suggests an underlying cognitive predisposition to gambling (and possibly comorbid addictions such as nicotine use), young people with this family history could undergo brief cognitive therapy focusing on these cognitive deficits (decision-making, working memory, cognitive inflexibility). In this way, we could prevent future development of more serious gambling problems as well as other addictive behaviors. Although speculative at this point, it could represent a targeted early intervention approach to gambling problems.

The domain of family history, however, may often be far more complex than a simple reflection of cognitive vulnerability. Having a parent with an SUD could be associated with childhood abuse or neglect, it could affect the kind of attachment developed by the child, and it may have associations with other psychosocial variables such as poverty, poor nutrition, and early life stress (Ahuja, Cunningham-Williams, Werner, & Bucholz, 2018; Calado, Alexandre, & Griffiths, 2017, 2018; Felsher, Derevensky, & Gupta, 2010). Any of these may in turn increase the probability of developing gambling problems as a means of coping with problems or emotions or alternatively lead to a need to over-control situations. Larger longitudinal studies are needed to parse out these complex components.

There are several limitations to this study. Participants were included in the study if they had some baseline level of gambling (having gambled at least five times in the preceding 12 months) and were not treatment seeking. Thus, these findings may not generalize to other less impulsive young adults in the community, or to clinical samples (including those in treatment). Second, we examined family history of SUDs as a unitary construct. Of course, it remains possible that family history of particular SUDs may have different associations from each other. However, our categorical approach (family history of any SUD versus not) is one that can easily be used in clinical practice whereas asking about history of a multitude of types of substance use disorders is challenging and time consuming for participants. The PLS approach had family history of addiction as the *Y* variable of interest, but we do not mean to suggest from this that *X* variables cause *Y*: rather, PLS was used as a method of constructing a model to maximally explain the relationships between two sets of variables, and the matrix with the larger set of variables is held in *X* by convention and for computational reasons. PLS does not indicate the direction of causality. We selected cognitive tests based on a review of the existing literature coupled with the need not to expose subjects to excessively long testing batteries; as such we did not quantify all domains and future work could examine other functions such as temporal discounting, Iowa Gambling Task performance, or executive planning. Another potential limitation is that we did not collect measures of fatigue during the cognitive testing sessions. However, in our experience cognitive testing of around 45 min is generally extremely well tolerated. Tasks were administered in a fixed order. Because medication use was not a reason for exclusion, the use of psychotropic medications could conceivably have affected cognitive performance in some subjects; we did not track medication use in the subjects, and so these findings may benefit from replication in subjects who are known not to be taking medications. Lastly, we did not differentiate between parental and sibling family history of SUDs.

In conclusion, this study found that family history of SUDs has a number of potentially clinically important associations in young adults who gamble, not only greater rates of addictive problems (alcohol, smoking, and gambling), but also relative impairments in some cognitive domains indicative of cognitive inflexibility and riskier decision-making. The latter may constitute trans-diagnostic markers that could run in families, acting as vulnerability markers for the development of different related addictive symptom domains.

## Funding sources

This study was supported by a grant from the National Center for Responsible Gaming. Dr. Chamberlain's role in this study was funded by a Wellcome Trust Clinical Fellowship (Reference 110049/Z/15/Z).

## Authors' contribution

Dr. Grant designed and conducted the study and drafted the manuscript. Dr. Chamberlain performed statistical analyses of the data and helped with writing the manuscript.

## Conflict of interest

Dr. Grant has received research grants from Promentis and Otsuka Pharmaceuticals. Dr. Grant receives yearly compensation from Springer Publishing for acting as Editor-in-Chief of the Journal of Gambling Studies and has received royalties from Oxford University Press, American Psychiatric Publishing, Inc., Norton Press, and McGraw Hill. Dr. Chamberlain consults for Promentis, and Ieso Digital Health. Dr. Chamberlain receives a stipend for his role as Associate Editor at Neuroscience and Biobehavioral Reviews; and at Comprehensive Psychiatry.
